# Comparing left atrial indices by CMR in association with left ventricular diastolic dysfunction and adverse clinical outcomes

**DOI:** 10.1038/s41598-021-00596-w

**Published:** 2021-10-29

**Authors:** James Nguyen, Jonathan Weber, Brittany Hsu, Rajasekhar R. Mulyala, Lin Wang, J. Jane Cao

**Affiliations:** 1 Department of Research, Department of Cardiovascular Imaging, The Heart Center, St Francis Hospital, 100 Port Washington Blvd, Roslyn, NY USA; 2 State University of New York at Stony Brook, Stony Brook, NY USA

**Keywords:** Cardiology, Medical research

## Abstract

Left atrial (LA) features are altered when diastolic dysfunction (DD) is present. The relations of LA features to the DD severity and to adverse outcomes remain unclear using CMR images. We sought to compare LA features including volumes, emptying fraction, and strains as predictors of left ventricular (LV) DD and adverse outcomes. We compared four groups including normal controls (n = 32), grade I DD (n = 69), grade II DD (n = 42), and grade III DD (n = 21). DD was graded by echocardiography following the current ASE guidelines. Maximum LA volume (LAV_max_), minimum LA volume (LAV_min_), and LA emptying fraction (LAEF) were assessed using CMR cine images. Phasic LA strains including reservoir, conduit, and booster pump strain were assessed by feature tracking. The outcome was a composite of hospital admissions for heart failure and all-cause mortality analyzed using Cox proportional hazard models. LAV_max_ and LAV_min_ were progressively larger while LAEF and LA strain measures were lower with worsening degree of DD (all *p* < 0.001). Among 132 patients with DD, 61 reached the composite outcome after on average 36-months of follow-up. Each of the LA parameters except for LA conduit strain was an independent predictor of the outcome in the adjusted Cox proportional hazard models (all *p* < 0.001). They remained significant outcome predictors after the model additionally adjusted for LV longitudinal strain. The AUC of outcome prediction was highest by LAEF (0.760) followed by LA reservoir strain (0.733) and LAV_min_ (0.725). Among all the LA features, increased LA volumes, reduced LAEF, reduced LA reservoir and booster pump strains were all associated with DD and DD severity. While LA strains are valuable, conventional parameters such as LAEF and LAV_min_ remain to be highly effective in outcome prediction with comparable performance.

Diastolic dysfunction (DD), a condition of impaired left ventricular (LV) relaxation, is a risk factor for heart failure (HF) and cardiac mortality irrespective of LV ejection fraction (LVEF)^1–3^. The associated increase in LV filling pressures results in dilation of left atrial (LA) size and loss of LA compliance and contractility^4,5^, which can be assessed using LA wall strain and LA emptying fraction (LAEF). Therefore, features of the LA are regarded as the barometer of DD. To date, many publications have addressed some LA features and have established compelling evidence that LA parameters are essential in the evaluation of DD^6–9^. However, few have compared the relative importance of their associations with DD and DD-related adverse outcomes. In addition, most of the published work is based on echo parameters. As Cardiovascular Magnetic Resonance Imaging (CMR) becomes increasingly utilized in the evaluation of cardiomyopathy or heart failure there is a need to better understand CMR based LA indices. In this study we sought to compare the associations of four LA parameters: minimum and maximum LA volume (LAV_min_ and LAV_max_), LAEF, and phasic LA strain (reservoir, conduit, booster pump) assessed by CMR, with DD, DD severity, and hazards of HF and mortality. We have also compared the hazards of LA features with that of LV longitudinal strain to demonstrate the relative importance of LA parameters.

## Methods

### Participants

This is a sub-study of a previously published larger study where participants underwent CMR and echocardiography within 7 days, between January 2007 and December 2015 at a single center^10^. The patient cohort (N = 132) was selected if there was evidence of DD, which was characterized by echocardiography based on the current ASE guidelines^11^. Of those, 66 were prospectively recruited for research and 66 were retrospectively analyzed from clinical studies. Patients were excluded if they were found to have history of atrial fibrillation, mitral stenosis, more than moderate mitral regurgitation, or prosthetic valve in the mitral position, which are conditions not supported by ASE guidelines for DD evaluation^11^. We also excluded subjects with indeterminate diastolic dysfunction grade. In addition to DD patients, normal controls were prospectively recruited who were free of cardiovascular history, major risk factors, and had both normal ECG and echocardiography. The vital signs and body surface area (BSA) for all subjects were collected at the time of CMR. Outcome data of hospitalized heart failure was extracted from electronic medical records of the health system consisting of 6 hospitals. The all-cause mortality information was obtained from National Death Index.

### Ethics approval and informed consent

This study was approved by the St. Francis Hospital Institutional Review Board. Informed written consent was obtained from all participants and research was performed in accordance with relevant guidelines and regulations.

### Imaging acquisition

#### Transthoracic echocardiography

Patients were scanned with a multi-frequency transducer ultrasound system (Philips IE 33, Andover, MA, USA). Comprehensive echocardiographic examination was performed using standard views. From the apical window, pulsed wave Doppler was used to record mitral inflow for 3–5 cardiac cycles at the level of the mitral valve annulus and at the mitral leaflets’ tips. Tissue Doppler was applied to record mitral annular velocities at the septal and lateral corners of the annulus. The resulting annular velocities by pulsed wave Doppler were recorded for 3 to 5 cardiac cycles at a sweep speed of 100 mm/s. Tricuspid regurgitation signals were recorded by continuous wave Doppler from multiple windows. Two-dimensional measurements were performed according to recommendations of the American Society of Echocardiography^11^ and indexed to body surface area. DD was assessed according to the algorithm from the recent ASE/EACI guidelines where mitral inflow early (E) and late (A) peak velocities, early diastolic annular myocardial longitudinal velocity (e′), tricuspid regurgitation and LA volume index were measured in order to determine the DD grade^12^.

#### CMR Imaging acquisition

All subjects underwent CMR on a 1.5 T scanner (Avanto, Siemens, Malvern, PA, USA) with an 8-element phased array surface coil. Balanced ECG-gated cine imaging of the long axis planes (2-, 3- and 4-chamber views) and a stack of 8–12 short axis planes (8 mm slice thickness, with 0 mm skip), starting from the mitral annulus, was acquired using balanced steady state free precession sequence with 30 phases per cardiac cycle. The average temporal resolution was 50 ms, with a field of view of 240 mm, flip angle of 70 degrees, repetition time (TR) of 3.1 ms and echo time (TE) 1.3 ms. All patients were in normal sinus rhythm at the time of imaging.

#### Post processing

**Figure 1:**
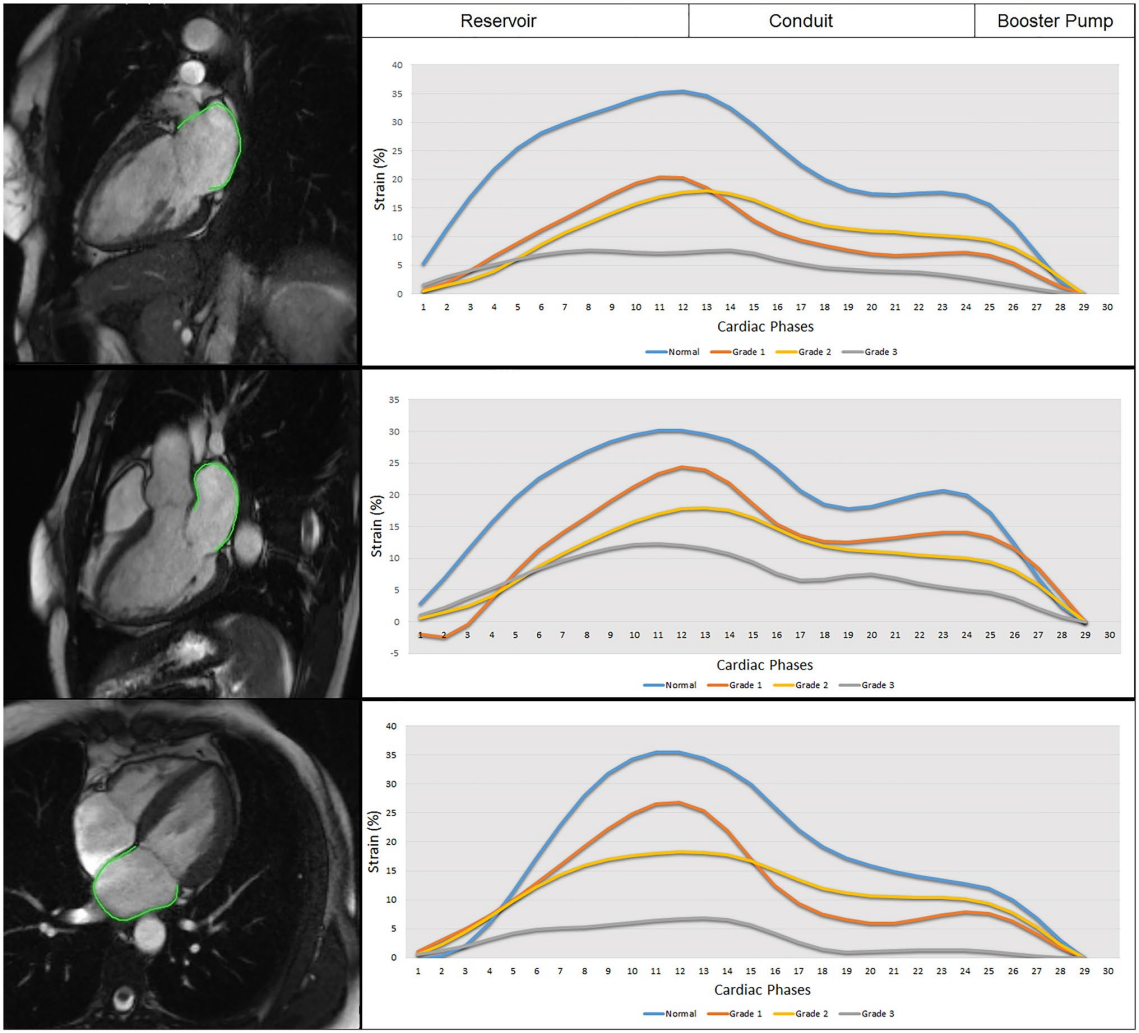
The left panel represents the respective 2-, 3-, 4-, chamber cine views from a normal control subject with the green feature tracking contours overlaying the left atrial wall. The right panels are the corresponding left atrial strain graphs obtained from each view as well as representative strain graphs from subjects with diastolic dysfunction grades 1, 2, and 3.

LA volume was analyzed following the area and length method^13^ using 2- and 4-chamber long axis cine images with commercial software (Circle Cardiovascular Imaging Inc, Calgary, Canada) and indexed to BSA. LA maximum volume (LAV_max_) was assessed at LV end systole and LA minimum volume (LAV_min_) at LV end diastole. Phasic LA strain was analyzed using feature tracking software (TomTec, Germany). LA endocardial contours were first drawn manually and propagated through cardiac phases along the 2-, 3-, and 4- chamber views. Manual adjustment was made when needed. Peak LA strains were assessed as the average peak strain values of the 3 longitudinal planes (Fig. 1). LV longitudinal strain was analyzed by feature tracking (Circle Cardiovascular Imaging Inc, Calgary, Canada) in order to capture the strain of full myocardial thickness. The epi- and endocardial contours were drawn manually on the end diastolic phase of the 2-, 3-, and 4-chamber cine images and propagated by the software to calculate 2D peak systolic longitudinal strain. All image analysis was performed by experienced operators.

### Statistical analysis

Continuous variables were described as mean (standard deviation) and count data as frequency (percent). Our primary exposures of interest were phasic LA strain (reservoir, conduit, and booster pump), LAEF, LAV_min_, and LAV_max_. Intra-observer reliability was assessed 6 months between the repeated measurements and reported with intra-class correlation coefficients (ICC). The association between our predictors and DD was assessed using area under the ROC curve (AUC) comparisons, and multinomial logistic regression. In these models, our covariates included age, gender, BSA, prevalent diabetes, hypertension, and smoking status chosen based on a disjunctive cause criterion^14^.

The association between our predictors of interest and a composite outcome of all-cause mortality and HF admission was assessed using Cox proportional hazards models. All-cause mortality data was obtained from the National Death Index and HF admissions based on electronic medical record review of the entire health care network. HF admissions were defined by an inpatient stay in a hospital setting with ICD 9 or ICD 10 diagnosis codes associated with congestive heart failure at the time of discharge. Individual Cox proportional hazards models were created for each primary exposure variable adjusting for age, gender, BSA (except for LAV_min_ and LAV_max_), diabetes, hypertension, and smoking status. The associations of LA parameters with the composite outcome were also examined using AUC comparisons. In addition, the association between E/A and E/a′ with composite outcome was assessed using AUC curves. Statistical analyses were performed using SAS v. 9.4 (Cary, NC, USA). 

## Results

### LA indices and diastolic dysfunction

**Figure 2:**
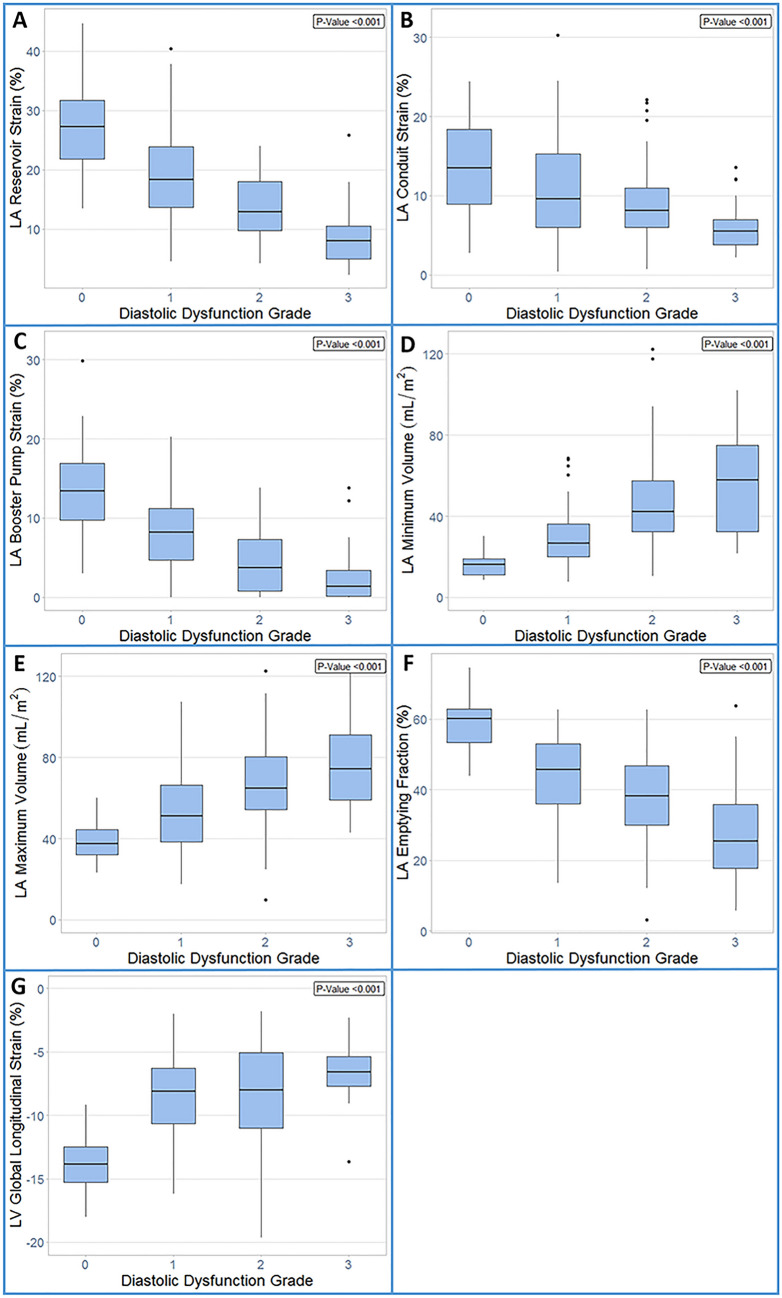
The graded change of left atrial reservoir strain (**A**), left atrial conduit strain (**B**), left atrial booster pump strain (**C**), left atrial minimum volume (**D**), left atrial maximum volume (**E**), left atrial emptying fraction (**F**), and left ventricular global longitudinal strain (**G**) in relation to the severity of left ventricular diastolic dysfunction. ANOVA with post-hoc Dunnett’s test yielded statistically significant differences between the means (*p* < 0.05) in each panel.

Our study subjects (n = 164) consisted of four groups: normal controls (n = 32), grade I DD (n = 69), grade II DD (n = 42), and grade III DD (n = 21). On average, patients with DD were 60 ± 14 years old and about half (N = 63) had advanced DD (grades II or III). Normal LVEF (> 50%) was present in 18 (14%) patients. Baseline characteristics of each DD grade are displayed in Table 1. Examples of phasic LA strains are shown in Fig. 1 where graded decreases of LA strains are shown from normal to grade 1, II and III diastolic dysfunction. Phasic LA strains and LAEF were progressively lower with increasing severity of DD (*p* < 0.001) (Fig. 2A–C, F; Table 2). Similarly, LV longitudinal strain was also progressively lower (Fig. 2G). Conversely, LAV_min_ and LAV_max_ were significantly higher with increasing severity of DD (*p* < 0.001) (Fig. 2D, E).

**Table 1:** Baseline characteristics* describing subjects with and without left ventricular diastolic dysfunction.

	Normal (N = 32)	Diastolic dysfunction grade I (N = 69)	Diastolic dysfunction grade II (N = 42)	Diastolic dysfunction grade III (N = 21)
Age (years)	44 (15)	57 (14)	65 (12)	62 (15)
Female (%)	11 (34)	8 (12)	15 (36)	10 (48)
Body surface area (m^2^)	1.93 (0.21)	1.46 (0.14)	1.41 (0.17)	1.42 (0.17)
Hypertension (%)	0 (0)	31 (45)	24 (57)	10 (48)
Diabetes mellitus (%)	0 (0)	15 (22)	13 (31)	3 (14)
Hyperlipidemia (%)	6 (19)	33 (49)	20 (48)	9 (43)
History of congestive heart failure (%)	0 (0)	33 (52)	26 (65)	16 (80)
Heart rate (bpm)	68 (13)	73 (15)	70 (21)	67 (27)
Systolic blood pressure (mmHg)	126 (19)	135 (20)	125 (37)	122 (34)
Diastolic blood pressure (mmHg)	72 (11)	80 (13)	68 (20)	72 (22)
Left ventricular ejection fraction (%)	57 (5)	37 (9)	44 (17)	31 (12)
Left ventricular end diastolic volume (mL/m^2^)	76 (16)	104 (37)	104 (46)	117 (48)
Left ventricular end systolic volume (mL/m^2^)	33 (9)	65 (32)	64 (48)	86 (50)
Left ventricular stroke volume (mL)	77 (24)	75 (20)	79 (23)	64 (17)
Right ventricular ejection fraction (%)	54 (6)	11 (20)	15 (23)	11 (17)
Right ventricular end diastolic volume (mL/m^2^)	72 (15)	69 (23)	74 (26)	77 (23)
Right ventricular end systolic volume (mL/m^2^)	33 (9)	37 (18)	40 (23)	48 (22)
Right ventricular stroke volume (mL)	71 (22)	65 (24)	68 (23)	60 (17)

*Described as mean (SD) or N (%).

**Table 2:** Distribution of left atrial strains and volumes across diastolic dysfunction categories.

	Normal diastolic function	Grade I diastolic dysfunction	Grade II diastolic dysfunction	Grade III diastolic dysfunction
	Mean (SD)	Mean (SD)	Mean (SD)	Mean (SD)
LA reservoir strain (%)	− 27.4 (8.13)	− 19.1 (8.01)	− 13.5 (5.42)	− 8.9 (5.77)
LA conduit strain (%)	− 13.9 (5.85)	− 10.4 (7.24)	− 9.3 (5.15)	− 6.2 (3.3)
LA pump strain (%)	− 13.6 (5.65)	− 8.7 (5.52)	− 4.2 (4.1)	− 2.8 (3.93)
LAV_min_ (ml/m^2^)	15.9 (5.23)	30.5 (14.84)	48.1 (24.48)	74.7 (66.51)
LAV_max_vol (ml/m^2^)	38.8 (10.13)	53.4 (18.81)	71 (26.5)	94.3 (66.86)
LAEF (%)	58.6 (8.21)	44.3 (12.04)	36.6 (13.95)	29.4 (17.61)
LV longitudinal strain (%)	− 13.9 (1.97)	− 8.4 (3.32)	− 8.6 (4.52)	− 6.5 (2.65)

A reproducibility analysis was performed on 15 randomly selected subjects. The Intra-observer reliability by ICC for LA indices were 0.99 (0.96, 1.00), 0.96 (0.89, 0.99), 0.92 (0.75, 0.98), and 0.6 (0.29, 0.79) for LAV_min_, LAV_max_, LAEF and LA reservoir strain respectively.

In order to examine the dose–response relationship, we created an adjusted, generalized multinomial logistic regression model to estimate the log-odds of the association between LA parameters of each DD grade compared with normal controls (Table 3). We observed significant dose–response relationship in LAV_min_, LAV_max_, LAEF, LA reservoir strain, and booster pump strain, but not in LA conduit strain. Using LA reservoir strain as an example, for every 1% decrease in LA strain the log-odds of a person having a higher grade of DD compared with normal control were 1.11 (CI 1.03–1.19), 1.25 (CI 1.14–1.38), and 1.50 (1.30–1.73) for grades I, II and III, respectively. In attempting to remove the potential confounding effect of LV strain on DD, we added LV longitudinal strain as a covariate to the previously described model and found that all exposures except LA conduit strain remained associated with worsening categories of DD (Table 3).

**Table 3:** The association between change in LA features and worsening categories of left ventricular diastolic dysfunction.

Left atrial measurement	Diastolic Dysfunction category	OR_ADJ_* (95% CL)	OR_ADJ_**(95% CL)
LA strain (reservoir) (%)	Normal	Reference	Reference
	I	1.11 (1.03, 1.19)	1.05 (0.96, 1.14)
	II	1.25 (1.14, 1.38)	1.20 (1.07, 1.34)
	III	1.50 (1.30, 1.73)	1.38 (1.17, 1.63)
LA strain (conduit) (%)	Normal	Reference	Reference
	I	1.05 (0.97, 1.14)	1.01 (0.90, 1.12)
	II	1.06 (0.97, 1.16)	1.01 (0.90, 1.13)
	III	1.24 (1.08, 1.42)	1.18 (0.99, 1.41)
LA strain (booster pump) (%)	Normal	Reference	Reference
	I	1.20 (1.08, 1.35)	1.15 (0.99, 1.34)
	II	1.47 (1.27, 1.70)	1.43 (1.19, 1.71)
	III	1.64 (1.35, 1.97)	1.48 (1.19, 1.85)
LAV_min_ (ml/m^2^)	Normal	Reference	Reference
	I	1.31 (1.13, 1.50)	1.33 (1.10, 1.61)
	II	1.37 (1.19, 1.58)	1.40 (1.16, 1.70)
	III	1.40 (1.21, 1.62)	1.43 (1.18, 1.73)
LAV_max_ (ml/m^2^)	Normal	Reference	Reference
	I	1.08 (1.03, 1.13)	1.13 (1.04, 1.23)
	II	1.12 (1.07, 1.17)	1.17 (1.08, 1.28)
	III	1.14 (1.08, 1.20)	1.19 (1.09, 1.30)
LAEF (%)	Normal	Reference	Reference
	I	1.16 (1.07, 1.24)	1.14 (1.04, 1.25)
	II	1.20 (1.11, 1.30)	1.19 (1.08, 1.31)
	III	1.24 (1.14, 1.35)	1.21 (1.09, 1.34)
LV longitudinal strain (%)	Normal	Reference	N/A
	I	1.61 (1.28, 2.03)	
	II	1.66 (1.31, 2.11)	
	III	1.99 (1.49, 2.65)	

OR_ADJ_ = adjusted odds ratio; 95% CL = 95% confidence limit; LAV_max_ = left atrial maximum volume; LAV_min_ = left atrial minimum volume; LAEF = left atrial emptying fraction.

*Adjusted for age, gender, BSA (except in models with LAV_max_ and LAV_min_), diabetes, hypertension, and smoking.

**Additionally adjusted for left ventricular global longitudinal strain.

### LA indices and composite clinical outcome

LA parameters were then examined as predictors of a composite outcome of HF admission and all-cause mortality. After a mean follow-up time of 38 ± 37 months, 61 (37%) patients reached the composite outcome due to 33 HF admissions and 28 deaths. No outcome events occurred in the normal controls. The outcome incidence was higher in more advanced DD: 45%, 63%, and 65% for grades I, II, and III, respectively (*p* < 0.001). After adjusting for age, BSA, gender, prevalent diabetes, hypertension, and smoking status, all LA parameters with the exception of LA conduit strain were independently associated with the composite outcome, and remained independently associated when the models were additionally adjusted for LV longitudinal strain (Table 4).

**Table 4:** The associations between LA features and increased hazards of clinical composite outcome.

	Unadjusted				Adjusted*				Adjusted**			
	HR	LL	UL	*p* value	HR	LL	UL	*p* value	HR	LL	UL	*p* value
LA strain (reservoir) per 1%	1.09	1.05	1.13	< 0.001	1.09	1.05	1.14	< 0.001	1.06	1.01	1.11	0.021
LA strain (conduit) per 1%	1.08	1.03	1.13	0.003	1.04	0.99	1.10	0.104	1.00	0.95	1.06	0.9
LA strain (booster pump) per 1%	1.09	1.03	1.14	0.001	1.11	1.05	1.17	< 0.001	1.09	1.02	1.16	0.007
LAV_min_ per 5 mL/m^2^	1.08	1.04	1.12	< 0.001	1.08	1.04	1.13	< 0.001	1.07	1.02	1.12	0.009
LAV_max_ per 5 mL/m^2^	1.08	1.03	1.12	< 0.001	1.08	1.03	1.14	0.002	1.08	1.03	1.13	0.003
LAEF per 1%	1.04	1.02	1.06	< 0.001	1.04	1.02	1.06	< 0.001	1.03	1.01	1.05	0.01
LV longitudinal strain per 1%	1.16	1.08	1.25	< 0.001	1.21	1.11	1.31	< 0.001	N/A			
LVEF per 1%	0.96	0.94	0.98	< 0.001	0.95	0.93	0.97	< 0.001	0.98	0.95	1.01	0.15
LVEDV per 5 mL/m^2^	1.05	1.02	1.08	< 0.001	1.07	1.04	1.10	< 0.001	1.03	1.00	1.07	0.080

HR = hazard ratio; LL = lower limit; UL = upper limit; LAV_max_ = left atrial maximum volume; LAV_min_ = left atrial minimum volume; LAEF = left atrial emptying fraction; LV = Left ventricle.

*Adjusted for age, gender, BSA (except in models with LAV_max_ and LAV_min_), diabetes, hypertension, and smoking status.

**Additionally adjusted for left ventricular global longitudinal strain.

In a post-hoc analysis with an intention to remove potential confounding from LV abnormalities we tested the independent associations of LA parameters with the composite outcome in models adjusting for LVEF and indexed LV end diastolic volume in addition to age, gender, BSA, diabetes and hypertension. We found that LA parameters remained to be independent predictors of outcome with HR 1.05 (1.00–1.10) for LAV_min_, 1.06 (1.00–1.11) for LAV_max_, 1.05 (1.00–1.10) for LA reservoir strain, 1.00 (0.95–1.05) for LA conduit strain and 1.07 (1.01–1.13) for LA booster pump strain. Overall, the results were similar to previously adjusted models.

**Figure 3:**
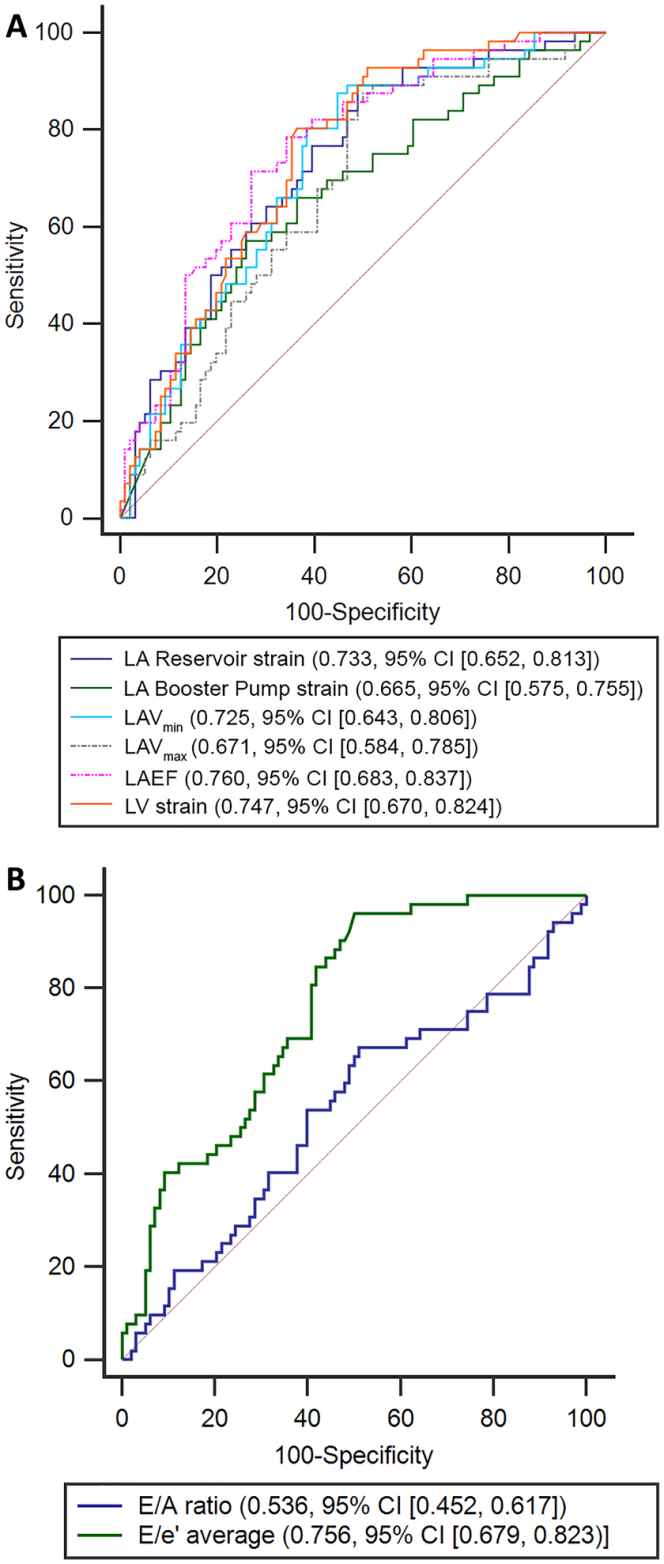
Receiver operating characteristic analyses of left atrial parameters (**A**) for predicting the composite clinical outcome as well as standard echocardiographic parameters for diastolic dysfunction (**B**) for comparison.

The associations of LA parameters with the composite outcome were additionally examined using AUC comparisons. The AUC was highest in LAEF (0.760) followed by LA reservoir strain (0.733) and LAV_min_ (0.725) (Fig. 3A). For reference purpose the AUC of LV longitudinal strain was 0.747. Additionally, we examined markers of diastolic dysfunction measured by echocardiography for reference including E/A ratio and averaged E/e′ ratio (Fig. 3B). The performance of E/e′ ratio (AUC 0.76) was similar to that of LAEF and reservoir LA strain but not E/A ratio (AUC 0.54). When comparing the AUC of LAV_max_ alone, the AUC of combined LAV_max_ and LAEF made an improvement in outcome prediction (AUC 0.77, *p* = 0.046) but combined LAV_max_ and LA reservoir strain did not (AUC 0.73, *p* = 0.28). Furthermore, combined LAV_min_ and LAEF (0.76) or LAV_min_ and LA reservoir strain (0.73) did not improve outcome prediction from LAV_min_ alone (*p* = 0.33 and *p* > 0.99, respectively).

## Discussion

In this study, we compared the relationships of LA parameters assessed by CMR including LAV_min_, LAV_max_, LAEF, and phasic LA strains with DD. Each LA parameter except LA conduit strain was independently associated with DD in a dose–response manner. Furthermore, each of the LA parameters except LA conduit strain was independently associated with the composite outcome of HF admission and all-cause mortality in the adjusted model including LV longitudinal strain or LVEF as a covariate.

Consistent with prior observations^15–18^, we found dose-responses between LAV (both LAV_max_ and LAV_min_) and the severity of DD. We have also confirmed prior reports that impaired LA function is closely associated with DD^19–22^ in a dose–response relationship in our study. Similarly, phasic LA strains also demonstrated similar dose–response relationships, confirming prior observations made from an echocardiography speckle-tracking study^23^.

While LA reservoir and booster pump strain are valuable, we found it is comparable at best to LAV_min_ and LAEF in predicting adverse clinical outcomes. The outcome prediction by LAV_max_ alone is not as strong as LAV_min_ and LAEF. But by combining it with LAEF, the prediction is significantly enhanced. Our findings highlights the strength of LAV_min_ in DD evaluation which is superior to widely used LAV_max_. Physiologically, LAV_min_ is directly modulated by LV filling pressure during LV end diastole as opposed to LAV_max_ which is predominantly influenced by the descending of mitral annulus thereby related to LV systolic function and LA compliance. Our collective findings suggest that LAV_min_ evaluation should be considered more favorably in the assessment of DD. It is also important to understand the relative importance of LA parameters in the context of other well-established imaging markers such as E/e′ and LV longitudinal strain in outcome prediction. We found that LAEF, the strongest outcome predictor of all LA indices including LA strain, has a comparable AUC from E/e′ and that from LV longitudinal strain. Given that LAEF correlates well with LV end diastolic pressure^18^ it is biologically plausible for it to predict clinical outcome effectively. Future research should examine the potential role of LAEF as an essential parameter in routine clinical evaluation.

It should be recognized that both LAV and LA strain evaluation are modality dependent. For instance, CMR has better spatial resolution than echocardiography rendering more accurate and reproducible LAV evaluation. In contrast, echocardiography has higher temporal resolution than CMR, hence providing a more favorable LA strain assessment. The technical differences may yield discrepancies in observations. That is why CMR specific investigations for LA in DD is essential despite the large body of literature using echocardiography. A recently published review article by Thomas et al.^24^ included a comprehensive list of studies that had investigated LAV and LA strain individually or collectively for their associations with DD. Most, if not all of the studies are echocardiography based and few, if any, use CMR. Additionally, we delineated the relative value of LAV_max_, LAV_min_, LAEF and phasic LA strain in DD and outcome prediction which has not been reported before. We have also established the associations of these LA indices with outcomes independent of LV longitudinal strain or LVEF. Of the LA parameters studied, LAV_min_ is easily attainable which allows LAEF to be calculated  from routine CMR or echocardiographic examination without the demand of additional post processing such as for LA strain. It is foreseeable to incorporate LAV_min_ and LAEF into future clinical algorithms for DD evaluation.

We recognize the limitations of our study. This is a pooled sample of both prospective and retrospective cohorts. The demographic data from the retrospective cohort was collected from electronic medical records and thereby subject to availability. CMR and echocardiography were performed within the same day for half of the DD patients but within 7 days for the other half. As DD diagnosis and grading were based on echocardiographic criteria, CMR evaluation that followed echocardiography was subject to the interval variation of hemodynamics, although there was no significant change reported during the interval. The DD patients consisted of both reduced and preserved EF. While the sample size is powered for outcome risk assessment we acknowledge the relatively small sample size originating from a single center clinical cohort. In addition, female gender is under represented. Similar to LV strain assessment, the value of LA strain is likely vendor dependent^25^. Therefore, the LA strain values generated from our normal subjects or from patients with DD cannot be directly compared with published studies using different vendors. Nonetheless, the relative importance of LA strain in our study that was compared with the normal controls is not subject to the variability of absolute value. It should also be noted that most of the feature tracking software is designed for LV strain evaluation and not for LA specifically. That is probably why the reproducibility of LA strain is only modest at best. However, our experience showed that the modern feature tracking program tracks the LA very well despite the thin wall. Tracking of the mobile mitral annulus is not always reproducible, and that seems to contribute to the variability. While the spatial resolution is excellent the temporal resolution of the CMR cine imaging is relatively low (about 50 ms) which may have compromised the accuracy of assessing the cardiac motion occurring within a short time frame such as LA boost pump strain. LA fibrosis evaluation by CMR can be valuable in patients with DD, but it was not performed in this study. While our electronic medical record allows us to capture HF admissions in the entire health system consisting of 6 hospitals, it is still possible that patients were admitted elsewhere, and therefore we may underestimate the scope of the outcomes. Lastly, this is a single center study and most of our patients are Caucasian. Future multicenter studies with diverse patient populations are warranted.

## Conclusion

LA features including LAV, emptying fraction, as well as LA reservoir and booster pump strains have dose–response relationships with DD severity. They are also independent predictors of HF admission and all-cause mortality. LAV_max_ is not a strong outcome predictor but the prediction can be enhanced significantly by combining it with LAEF. While LA strains are valuable, conventional parameters such as LAEF and LAV_min_ remain highly effective in outcome prediction with comparable performance.

## Data Availability

The datasets used and/or analysed during the current study are available from the corresponding author on reasonable request.

## Abbreviations

DD: Diastolic dysfunction
LV: Left ventricular
HF: Heart failure
LA: Left atrial
CMR: Cardiac magnetic resonance imaging
LAV_min_: Left atrial minimum volume
LAV_max_: Left atrial maximum volume

## References

[1] Owan TE, Hodge DO, Herges RM, Jacobsen SJ, Roger VL, Redfield MM (2006). Trends in prevalence and outcome of heart failure with preserved ejection fraction. N. Engl. J. Med..

[2] Vasan RS, Benjamin EJ, Levy D (1995). Prevalence, clinical features and prognosis of diastolic heart failure: An epidemiologic perspective. J. Am. Coll. Cardiol..

[3] Steinberg BA, Zhao X, Heidenreich PA, Peterson ED, Bhatt DL, Cannon CP, Hernandez AF, Fonarow GC (2012). Trends in patients hospitalized with heart failure and preserved left ventricular ejection fraction: Prevalence, therapies, and outcomes. Circulation.

[4] Santos AB, Roca GQ, Claggett B, Sweitzer NK, Shah SJ, Anand IS, Fang JC, Zile MR, Pitt B, Solomon SD, Shah AM (2016). Prognostic relevance of left atrial dysfunction in heart failure with preserved ejection fraction. Circul. Heart Failure.

[5] Freed BH, Daruwalla V, Cheng JY, Aguilar FG, Beussink L, Choi A, Klein DA, Dixon D, Baldridge A, Rasmussen-Torvik LJ, Maganti K (2016). Prognostic utility and clinical significance of cardiac mechanics in heart failure with preserved ejection fraction: Importance of left atrial strain. Circul. Cardiovasc. Imaging.

[6] Imai M, Ambale Venkatesh B, Samiei S, Donekal S, Habibi M, Armstrong AC, Heckbert SR, Wu CO, Bluemke DA, Lima JA (2014). Multi-ethnic study of atherosclerosis: Association between left atrial function using tissue tracking from cine MR imaging and myocardial fibrosis. Radiology.

[7] Pritchett AM, Mahoney DW, Jacobsen SJ, Rodeheffer RJ, Karon BL, Redfield MM (2005). Diastolic dysfunction and left atrial volume: A population-based study. J. Am. Coll. Cardiol..

[8] Kurt M, Wang J, Torre-Amione G, Nagueh SF (2009). Left atrial function in diastolic heart failure. Circul. Cardiovasc. Imaging.

[9] Morris DA, Gailani M, Pérez AV, Blaschke F, Dietz R, Haverkamp W, Özcelik C (2011). Left atrial systolic and diastolic dysfunction in heart failure with normal left ventricular ejection fraction. J. Am. Soc. Echocardiogr..

[10] Wang L, Singh H, Mulyala RR, Weber J, Barasch E, Cao JJ (2020). The association between left ventricular diastolic dysfunction and myocardial scar and their collective impact on all-cause mortality. J. Am. Soc. Echocardiogr..

[11] Nagueh SF, Smiseth OA, Appleton CP, Dokainish H, Edvardsen T, Flachskampf FA, Gillebert TC, Klein AL, Lancellotti P, Marino P, Oh JK (2016). Recommendations for the evaluation of left ventricular diastolic function by echocardiography: An update from the American Society of Echocardiography and the European Association of Cardiovascular Imaging. Eur. Heart J. Cardiovasc. Imaging.

[12] Mitchell C, Rahko PS, Blauwet LA, Canaday B, Finstuen JA, Foster MC, Horton K, Ogunyankin KO, Palma RA, Velazquez EJ (2019). Guidelines for performing a comprehensive transthoracic echocardiographic examination in adults: Recommendations from the American Society of Echocardiography. J. Am. Soc. Echocardiogr..

[13] Maceira AM, Cosín-Sales J, Roughton M, Prasad SK, Pennell DJ (2010). Reference left atrial dimensions and volumes by steady state free precession cardiovascular magnetic resonance. J. Cardiovasc. Magn. Reson..

[14] VanderWeele TJ (2019). Principles of confounder selection. Eur. J. Epidemiol..

[15] Morris DA, Belyavskiy E, Aravind-Kumar R, Kropf M, Frydas A, Braunauer K, Marquez E, Krisper M, Lindhorst R, Osmanoglou E, Boldt LH (2018). Potential usefulness and clinical relevance of adding left atrial strain to left atrial volume index in the detection of left ventricular diastolic dysfunction. JACC Cardiovasc. Imaging..

[16] Tsang TS, Abhayaratna WP, Barnes ME, Miyasaka Y, Gersh BJ, Bailey KR, Cha SS, Seward JB (2006). Prediction of cardiovascular outcomes with left atrial size: Is volume superior to area or diameter?. J. Am. Coll. Cardiol..

[17] Russo C, Jin Z, Homma S, Rundek T, Elkind MS, Sacco RL, Di Tullio MR (2012). Left atrial minimum volume and reservoir function as correlates of left ventricular diastolic function: Impact of left ventricular systolic function. Heart.

[18] Posina K, McLaughlin J, Rhee P, Li L, Cheng J, Schapiro W, Gulotta RJ, Berke AD, Petrossian GA, Reichek N, Cao JJ (2013). Relationship of phasic left atrial volume and emptying function to left ventricular filling pressure: A cardiovascular magnetic resonance study. J. Cardiovasc. Magn. Reson..

[19] Sanchis L, Andrea R, Falces C, Lopez-Sobrino T, Montserrat S, Perez-Villa F, Bijnens B, Sitges M (2016). Prognostic value of left atrial strain in outpatients with de novo heart failure. J. Am. Soc. Echocardiogr..

[20] Ramkumar S, Yang H, Wang Y, Nolan M, Negishi T, Negishi K, Marwick TH (2017). Association of the active and passive components of left atrial deformation with left ventricular function. J. Am. Soc. Echocardiogr..

[21] Georgievska-Ismail L, Zafirovska P, Hristovski Z (2016). Evaluation of the role of left atrial strain using two-dimensional speckle tracking echocardiography in patients with diabetes mellitus and heart failure with preserved left ventricular ejection fraction. Diab. Vasc. Dis. Res..

[22] Kowallick JT, Kutty S, Edelmann F, Chiribiri A, Villa A, Steinmetz M, Sohns JM, Staab W, Bettencourt N, Unterberg-Buchwald C, Hasenfuß G (2014). Quantification of left atrial strain and strain rate using Cardiovascular Magnetic Resonance myocardial feature tracking: A feasibility study. J. Cardiovasc. Magn. Reson..

[23] Singh A, Addetia K, Maffessanti F, Mor-Avi V, Lang RM (2017). LA strain for categorization of LV diastolic dysfunction. JACC: Cardiovasc. Imaging.

[24] Thomas L, Marwick TH, Popescu BA, Donal E, Badano LP (2019). Left atrial structure and function, and left ventricular diastolic dysfunction: JACC state-of-the-art review. J. Am. Coll. Cardiol..

[25] Cao JJ, Ngai N, Duncanson L, Cheng J, Gliganic K, Chen Q (2018). A comparison of both DENSE and feature tracking techniques with tagging for the cardiovascular magnetic resonance assessment of myocardial strain. J. Cardiovasc. Magn. Reson..

